# Health and well-being literacy initiatives focusing on immigrant communities: an environmental scan protocol to identify "what works and what does not"

**DOI:** 10.1186/s13643-020-01340-7

**Published:** 2020-04-20

**Authors:** Tanvir C. Turin, Nashit Chowdhury, Mahzabin Ferdous, Marcus Vaska, Nahid Rumana, Rudra Dahal, Nafiza Rahman, Mohammad Z. I. Chowdhury

**Affiliations:** 1 Department of Family Medicine, Cumming School of Medicine, University of Calgary, G012F, Health Sciences Centre, 3330 Hospital Drive NW, Calgary, Alberta T2N 4N1 Canada; 2 Department of Community Health Sciences, Cumming School of Medicine, University of Calgary, 3280 Hospital Drive NW, Calgary, AB T2N 4Z6 Canada; 3 Knowledge Resource Service, Tom Baker Cancer Centre, Alberta Health Services, 1331-29 St. NW, Calgary, AB T2N 4N2 Canada; 4 Sleep Center, Foothills Medical Center, University of Calgary, 1403-29 St NW, Calgary, AB T2N 2TN Canada; 5 Community Based Citizen Researcher, Calgary, AB Canada

## Abstract

**Introduction:**

Most of the major cities in the developed western countries are characterized by an increasing multiculturalism brought by the immigrant population. The immigrant communities face challenges in the new environment with their health and wellness related unmet needs. It is imperative to find sustainable ways to empower these diverse communities to champion their health and wellness. Community-based health and wellness literacy initiatives (CBHWLI) focusing on immigrant communities can be an important step towards citizen empowerment in this regard. The aim of the present environmental scan is to identify the key factors that might impact a CBHWLI in immigrant communities in Canada in order to facilitate the process in practice and identify the competencies and training required for its implementation.

**Methods:**

This study will gather information from existing literature and online sources as well as will capture expert and lay perspectives on the factors that can impact the effectiveness and sustainability of CBHWLIs through conducting a comprehensive environmental scan: (i) a systematic scoping review of published literature and grey literature, (ii) a comprehensive Internet search, (iii) key informant interviews, and (iv) community consultation. Specific methodological and analytical frameworks will guide each step.

**Ethics and dissemination:**

This study is the first step in establishing a practical base for developing CBHWLI implementation research. Once the initial findings have been generated, the second step will involve inviting experts to provide their input. We first plan to disseminate the results of our scoping review and Internet scan through meetings with key stakeholders, to be followed by journal publications and conference or workshop presentations. Ethical approval is not required for the scoping review or Internet scan; however, approval to conduct interviews with key informants and community consultations in the second stage of the study will be sought from the Conjoint Health Research Ethics Board.

## Strengths and limitations of this study

- Multiple information collection comprehensive strategies covering extensive information sources will be employed.
- The inclusion criteria include both academic and grey literature to ensure comprehensiveness but will be limited to publications in English.

## Introduction

Health literacy is a critical determinant of health that largely impacts both the individual and community health and wellness [1]. It helps individuals to improve preventive practices, to enhance patient empowerment and to reduce health disparities among different social-cultural groups [2, 3]. Furthermore, current trends in healthcare practice increasingly emphasize the engagement of patients/community members in making health-related decisions and managing their own health [4]. Health literacy is more than reading and understanding health information; rather, “it is the ability to access, comprehend, evaluate and communicate the information as a way to promote, maintain, and improve health in a variety of settings across one’s lifespan” [2]. Low health literacy may lead to delayed diagnosis, poor administration of the prescribed medication, higher morbidity and mortality rates as well as increased hospitalization rates and healthcare expenditures [5].

The major urban centers all over the world, especially in the developed western countries, are characterized by an increasing multiculturalism brought by the immigrant population as a consequence of the accelerated globalization process [6]. The immigrants tend to be less knowledgeable about the regulations and customs of their new host country’s health system [7]. They also face difficulty navigating health services, understanding basic health and wellness information, and concepts of common diseases, as well as their diagnostic and treatment procedures [8]. The dual challenges of limited health literacy and cultural differences are likely to increase an expanding and increasingly diverse population in countries like Canada, the USA, or some European countries where immigrant settlement happens in large numbers. It is thus imperative to engage these diverse communities towards improvement initiatives to their health and wellness. Meaningfully engaged and sustainable health and wellness literacy initiatives focusing on immigrant communities would be a very important step towards community empowerment for health and wellness in this regard. This is especially important because poor health literacy is consistently reported to be associated with adverse health behaviors and outcomes and has been reported to partially explain racial disparities in some outcomes [9]. However, the availability, efficacy, and extent of health literacy initiatives specific to the immigrant populations have not been systematically summarized. A comprehensive understanding of the health and wellness literacy initiatives focusing on the immigrant population will help us to outline the nature and outcomes of those initiatives as well as their weaknesses and strengths in order to devise a working structure to improve health and wellness literacy among immigrant individuals through community initiatives.

The aim of the present environmental scan is to identify the key factors that might impact a community-based health and wellness literacy initiatives (CBHWLI) in immigrant communities in Canada in order to facilitate the process in practice and identify the competencies and training required for its implementation.

## Objective

The present protocol for an environmental scan aims to identify the key opportunities and challenges for implementing CBHWLIs focusing on immigrant communities. More specifically, the objective of the present protocol is to identify the key factors that might facilitate the process and outcomes of a health literacy program in practice as well as to identify competencies and training required for its effective implementation by overcoming the challenges.

### Community involvement

To prepare the research idea and develop this environmental scan proposal, we have partnered actively with community champions and citizen researchers at the community level from the very beginning. Community champions are the persons who proactively take actions on community issues or act as an intermediary between the grassroots community and other entities; including government, foundations, service providers, academia, and other community organizations. Our citizen researchers are the community members who are actively contributing to our program of research through participation in different phases of our research projects; including research question contextualization, guiding cultural competencies of our proposed methodologies, recruitment and data collection, making sense of the analyzed data, contributing in knowledge mobilization. We had regular discussions with them to get their input, which contributed significantly to shaping our logic model (Table 1) and guiding questions (Table 2). They will also be involved in the interpretation of synthesized information, recruitment of potential stakeholders for interviews and will actively guide executing community-engaged focus group discussions. They also will be at the forefront of our knowledge translation and dissemination initiatives. They have agreed to guide us in choosing information to share, the process to share, as well as use their community influence to mobilize towards the extended community. Moreover, this will be a learning opportunity for them to gain insight into the methodological aspects of comprehensive information searching through an environmental scan.

**Table 1:** Logic map for the proposed environmental scan on community-based health and wellness literacy initiatives (CBHWLI)

Steps	Aim	Inputs	Activities	Key circles	Outputs	Outcomes
Scoping review of published documents to gather published information	• Summarize the existing literature regarding health & wellbeing literacy • Determine the research gaps	Scoping review • Grey literature review	• Preliminary information search • Brainstorming session to develop search terms and databases • Refine search strategy	• Research team • Librarian • Citizen researcher from the community	• Environmental scan report • Summary of the existing literature	• Obtain a clear sense of available research • Increased knowledge on the topic
Internet scan to review the existing health and wellbeing literacy initiatives	• Identify the existing CBHWLIs • Inventory the mode of operations and logistical requirements	• Comprehensive Web search	• Preliminary information search • Brainstorming session to develop search terms, and databases • Refine search string, strategy	• Research team • Communication experts • Citizen researcher from the community	• Environmental scan report • Identify existing mode of operations • Identify potential logistical needs	• Obtain a clear sense of available models of CBHWLIs • Increased knowledge on the topic
Key informant interviews to gather professional information from unpublished sources	• Gather a full range of perspectives on CBHWLIs	• Key informant interviews: a. Identifying key informants through stakeholder analysis b. Engagement (recruitment) c. Interviews d. Data transcription e. Data analysis and presentation	• Collect feedback from stakeholders • Establish partnerships • Share findings from the literature review with stakeholders	• Research team • Stakeholders • Community champions • Citizen researcher from the community	• Environmental scan report • Identify key issues and priorities	• Receive practical comments from the stakeholders to incorporate in the next steps of full project development • Community champions have an increased understanding about the CBHLI • Their points of view are noted for further exploration into having them as a partner, if needed
Community engagement for lay perspective	• Understand community perspectives on CBHWLIs • Understand community’s knowledge, attitude and practice towards CBHWLIs • Acquire in-depth narratives	• Focus group discussions: a. Identify participants b. Engagement (recruitment) c. Focus group discussions d. Data transcription, analysis, and presentation	• Engagement • Conduct focus groups with participant groups • Complete survey questionnaires with participant groups • Data analysis • Update project stakeholders on findings of the focus groups and surveys	• Research team • Community champions • Citizen researcher from the community	• Environmental scan report • Identify community perspectives on the possible CBHWLI	• Identify community members’ perceptions that may influence CBHWLI initiation • The research team increases their knowledge of how to communicate effectively with community people • Community participants increase their knowledge, skills, and confidence in engaging in a CBHWLI initiative

**Table 2:** Guiding questions for conducting the environmental scan

Current evidence on health and wellness literacy initiatives	Gaps in knowledge about health and wellness literacy initiatives	Basics on health and wellness literacy initiatives formation process
What exactly is health and wellness literacy initiative?	What are the key uncertainties regarding a health and wellness literacy initiative in terms of impact?	What types of health and wellness literacy initiatives exist?
What health and wellness literacy initiatives have shown previous benefit?	What are the ongoing studies on health and wellness literacy initiatives?	What are the benefits and drawbacks to each health and wellness literacy initiative?
What are the previous benefits or drawbacks of health and wellness literacy initiatives?	What populations are currently receiving health and wellness literacy initiatives?	What aspects of health and wellness literacy initiatives are likely to make them more or less effective?
In what populations have health and wellness literacy initiatives been implemented and studied?	What aspects of health and wellness literacy initiatives matter to different stakeholders?	For what aspect of a population was a health and wellness literacy initiative developed? And was the impact measured?

## Methods

**Fig. 1:**
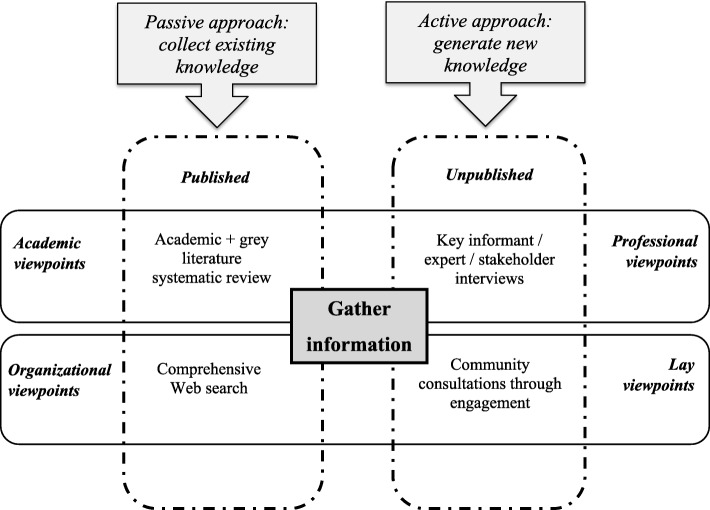
Information gathering schema: viewpoint capturing and sources of evidence

Environmental scan is an important information gathering methodology around an issue, organization, or intervention which can be employed in the public health domain to gather evidence for improving policy and practice [10]. The environmental scan will use both passive and active information collection approaches using four key activities [10] (Fig. 1): (i) a systematic scoping review (published and grey literature), (ii) a comprehensive Internet search, (iii) key informant interviews, and (iv) community consultation through engagement. A detailed logic map of the process is presented in Table 1. Table 2 shows the guiding questions for conducting the environmental scan. The following sections describe the protocol and provide methodological details.

### Systematic scoping review

As the first step, a scoping review will allow us to systematically map the available literature on the topic and identify the gaps in research. Moreover, scoping reviews form the preliminary step towards full syntheses [11, 12]. Therefore, using predetermined keywords, we will undertake a scoping review of electronic databases searching for journal articles, supplemented with an investigation of the grey literature. This approach will follow the review methodology outlined by Arksey and O’Malley [11] and Levac, Colquhoun, and O’Brien [13]. We will also gather information from secondary sources, such as publicly available information, to supplement the results obtained from the active approach discussed below.

#### Identifying the research question

An effective scoping review requires a research question situated within a specific area, but it must also remain broad so as not to exclude potentially useful literature [11]. For this review, we posed the following non-limiting questions: What is known about CBHWLI and which aspects of CBHWLIs matter to different stakeholders as identified by original research? What aspects of CBHWLIs are likely to make them effective? What are the key uncertainties regarding CBHWLIs?

#### Study selection eligibility/inclusion criteria

As mentioned, the purpose of this scoping review is to identify all relevant literature using wide definitions of terms to ensure broad coverage. Hence, we will not evaluate the methodological quality of studies guided by a strict research question with narrow inclusion and exclusion criteria, as in systematic reviews. We will not restrict studies based on country of origin or date of publication for all potentially relevant citations to be retrieved but will exclude all studies focusing on a single disease-related patient population. We will select studies published in the English language only.

#### Comprehensive systematic search

A pragmatic approach to literature searching will be taken, encompassing both traditional systematic search methods and a grey literature review to identify relevant documents. The grey literature review will be conducted to gather information from unpublished or in-progress research and also to learn what kinds of CBHWLIs are publicly available to communities. This information will be used to detail existing CBHWLIs and, in conjunction with data from other sources, to identify gaps in the evidence about such initiatives. We will conduct a comprehensive search [14] of literature repositories for relevant studies based on sets of appropriate keywords (see Table 3 for the list of databases to be searched).

**Table 3:** List of databases searched to identify literature for this synthesis

Published articles	Grey literature
*Health sciences:* • MEDLINE (Ovid) • EMBASE • PsycINFO • EBM Reviews • HealthSTAR • PubMed • PubMed Central • CINAHL • MEDLINE (Ebsco) *Social sciences:* • Psychology & Behavioral Sciences Collection • Social Science Data Archive • SocIndex with FullText • Sociological Abstracts • Social Work Abstracts *Multi-disciplinary:* • Web of Science • Education Research Complete • ERIC • Urban Studies Abstracts • Scopus • Canadian Research Index • LegalTrac • International Political Science Abstracts • PAIS Index • Leading Practices Database • Business Source Complete	*Academic-focused search engines:* • Google Scholar *Repositories/theses:* • ProQuest (theses and dissertations) • OAISter (WorldCat) *Health sciences:* • Health Sciences Online (HSO) • Turning Research into Practice (TRIP) • Canadian Institutes of Health Research (CIHR) • Canadian Institute for Health Information (CIHI) • Canadian Public Health Association (CHPA) • Public Health Agency of Canada (PHAC) • Public Health Ontario • Health Quality Ontario Health Canada • National Institutes of Health (NIH) • World Health Organization (WHO) • National Health Services (NHS) • Alberta Health Services (AHS) Insite • National Health and Medical Research Council, Australia • European Commission • U.S. Department of Health (Health.gov) • Centers for Disease Control and Prevention (CDC) • Global Health Literacy Academy *Social sciences:* • International Federation of Social Science Organizations (IFSSO) • Federation of Data Organizations for Social Science (IFDO) • Consortium of Social Science Associations (COSSA) • Organization for Social Science Research in Eastern and Southern Africa (OSSREA) • International Organization of Social Sciences and Behavioral Research (IOSSBR) • ABC Life Literacy Canada *Other:* • Canadian Public Legal Education (CPLE) organizations

#### Published literature

Literature searches will be conducted within the bibliographic databases presented in Table 3 to identify articles for the review. An experienced librarian, a research team member, will oversee the development and execution of the database search strategies, which include a predefined list of keywords and medical subject heading (MeSH) terms (Table 4). This method adheres to the Cochrane Collaboration approach towards systematic searching, whereby the controlled vocabulary (MeSH terms) is combined with keyword searching [14, 15]. We will also review the reference lists of all the selected papers to identify additional studies that should be considered for inclusion.

**Table 4:** Search terms in detail

*Search terms for* health literacy:	
Health literacy [keyword, MeSH]; (health OR wellness OR wellbeing) [Keyword]; literacy [keyword, MeSH]; well* adj3 literacy; Patient Education as Topic [MeSH]; Health Education [keyword, MeSH];”health knowledge” [Keyword]; Information Literacy [keyword, MeSH]; Prenatal Education [keyword, MeSH]; Patient Education Handout [keyword, MeSH]; Health Education, Dental [keyword, MeSH]; Consumer Health Information [keyword, MeSH]; Health Information [keyword, MeSH];;	
*Search terms for behavioral traits/actions of health literacy*	
Health knowledge, attitudes, practice [MeSH]; health promotion [keyword, MeSH]; health numeracy [keyword]; patient participation [MeSH]; patient empower* [keyword]; health empower* [keyword]; health behaviour [keyword]; health behavior [keyword, MESH[; self-administration [keyword, MESH]; decision making [keyword, MeSH]; informed decision [keyword]; informed consent [keyword, MeSH]; health-related decision making [keyword]	
*Search terms for immigrant:*	
Immigrant* [keyword]; Immigrants [MeSH]; emigrant* [keyword]; alien* [keyword]; “emigrants and immigrants” [MeSH]; Undocumented immigrant* [keyword, MeSH]; Newcomer* [keyword]; Refugee* [keyword, MeSH]; asylum [keyword]; asylum seeker [keyword]; displaced [keyword]; resettle [keyword]; Humanitarian [keyword]; entrant [keyword]; settle [keyword]; displaced person [keyword]; displaced population [keyword]; “internally displaced person” [keyword]; “war population” [keyword]; “forced migra*” [keyword]; “refugee camp*” [keyword]; Refugee Camps [MeSH]	
*Search string formation:*	
(“health literacy” OR “patient education as a topic” OR “health education” OR “health knowledge” OR “information literacy” OR “prenatal education” OR “patient education handout” OR health education, dental OR “consumer health information” OR “health information” OR “health promotion” OR “health numeracy” OR “patient empower*” OR “health empower*” OR “health promotion” OR “health knowledge, attitudes, practice” OR “patient participation” OR “health behaviour” OR “health behavior” OR “self-administration” OR “decision making” OR “informed decision” OR “informed consent” OR “health-related decision making” OR ((health OR wellness OR well-being OR wellbeing) AND (literacy OR education OR information)) OR ((access* OR seek* out OR obtain* OR find* OR gain*) AND (“health information” OR “consumer health information”)) OR ((understand* OR perceive* OR perception OR comprehen*) AND (“health information” OR “consumer health information”)) OR ((apprais* OR assess* OR evaluat* OR interpret* OR judge* OR apply*) AND (“health information” OR “consumer health information”))) AND (immigrant* OR emigrant* OR alien* OR “undocumented immigrant*” OR newcomer* OR refugee* OR asylum OR “asylum seeker” OR displaced OR resettle OR humanitarian OR entrant OR settle OR “displaced person” OR “displaced population” OR “internally displaced person” OR “war population” OR “forced migra*” OR “refugee camp*”)	

#### Grey literature

For grey literature, our search strategy will include electronic institutional repositories, national and provincial, and international professional and government websites, as well as established search engines [16] (see Table 3 for a complete search database list). Unpublished dissertations and theses will be sought using the ProQuest Dissertations and Theses database. Information about in-progress research projects will be gathered from Health Services Research Projects in Progress (HSRProj).

From the outset, it was identified that this review would need to capture the grey literature in addition to peer-reviewed materials. As such, a pragmatic approach to literature searching was taken, encompassing both traditional systematic search methods and extensive consultation to identify relevant documents. Searches of the electronic databases, EBSCO Host, CINAHL, and Web of Science were conducted. In addition, the Google search engine was used to identify the websites of key international professional organizations and locate relevant materials. Search terms included competence, competency standards, competency statement, professional practice combined with PHC, general practice, community, office nursing and nurs*. Key stakeholders were individually emailed and asked to identify any materials they knew to be relevant. The reference lists of retrieved materials were searched for additional sources. Given the significant changes occurring in the PHC environment, the search was limited to items published since 2000. Due to resource constraints that precluded translations, only English language materials were included.

#### Identifying relevant studies/article screening

**Fig. 2:**
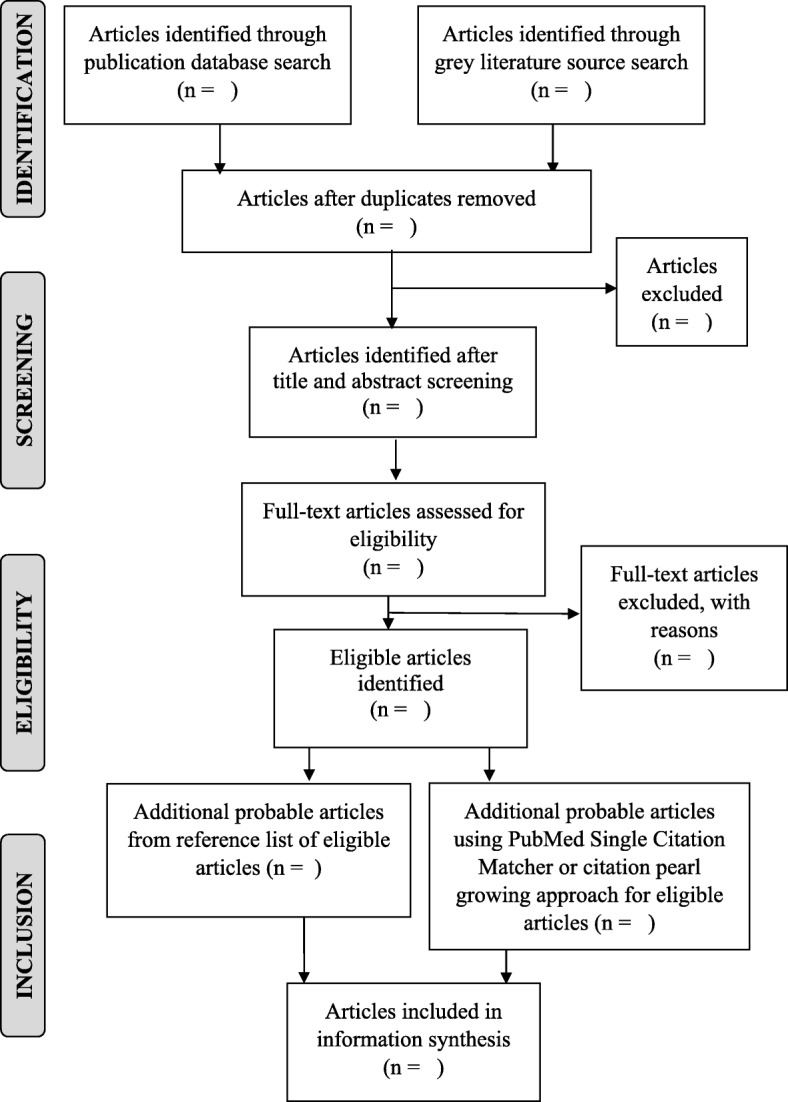
Flow diagram of search and selection process for the systematic scoping review

All titles retrieved through the comprehensive search will be screened for relevance. RefWorks software (ProQuest, LLC, Ann Arbor, MI, USA) will be used to manage retrieved articles. We will follow a two-step screening process: (a) title-abstract review and (b) full-text review (Fig. 2). In the first screening step, two researchers will independently review the title and abstract for each paper to document and decide whether it should be included or excluded. Abstracts will be classified as relevant, potentially relevant or not relevant. Abstracts that do not provide enough information on outcomes to determine eligibility will be included for further review. Full texts will be obtained of the abstracts that meet eligibility criteria and will be read, reviewed, and re-examined for relevance. During the second screening step (full-text screening), two researchers will independently review the full text of the included papers after the initial screening to determine eligibility. If no agreement is reached between the two researchers, a third researcher will arbitrate.

#### Data abstraction and data charting

Pertinent information will be abstracted from the included studies. Two trained research team member will extract the information in a predetermined abstraction tool. They will work concurrently and meet intermittently to compare their results. Information will be extracted on the citation, study location, study objective, how the initiative was established, the main outcome variables, how the outcome variables were measured, etc (Table 5). The charted data will form the basis for the analysis and will follow the narrative tradition’s “descriptive-analytical method,” which involves collecting standard information from each research report and applying a common analytic framework to all included studies. Excel (Microsoft Corporation, Redmond, WA, USA) will be used to build a database from the extracted information for result synthesis.

**Table 5:** Data extraction sheet for information abstraction from the identified studies

Reviewer	###
Review date	###
Author(s)	###
Year	###
Title	###
Journal	###
Publication type	
Study design	###
Study objective	###
Study country	###
Study setting	###
Study population	###
Sample size	###
CBHWLI definition/scope/context	###
CBHWLI methodology	
CBHWLI target population	###
CBHWLI focus/topic	###
CBHWLI location/society	###
CBHWLI structure	###
Outcome measured	
Data collection method	###
Challenges mentioned	### ###
Facilitators mentioned	### ###
Impacts mentioned	### ###
Strengths mentioned	### ###
Limitations mentioned	### ###
Comments	

#### Summarizing and reporting results

Our review is intended to develop a description of what research exists and create a broad view of research in the topic area. Data will be collected, synthesized, and presented using summary tables to highlight the current state of evidence regarding CBHWLIs. The research team will review the data summary tables to determine the optimal use and presentation of information from these tables in a manuscript suitable for publication. We will develop a standard format for summarizing and presenting descriptive and methodological information and outcomes of included studies. This will include recording dimensions (methodology, description of study objectives [focus, target audience]), any definitions offered (definitions of data, literacy, education, etc.), and any findings and opinions related to activities intended to enable the use of knowledge in practice by health professionals (Table 5). The extracted information will be compared, and patterns will be recorded as they become apparent.

### Comprehensive Internet search

Internet searching is valuable for identifying a variety of information in non-academic domains including program descriptions, reports or policy briefs, evaluation results, public perception, and opinions. To understand what information on CBHWLIs are publicly available, we will use the established search engines to identify the websites of key national and international professional organizations to assist in locating relevant material. We will also consider several important factors while searching the Internet. For example, suitable search engines must be powerful enough to handle complex queries, because web documents are not indexed with keywords in a controlled vocabulary [17]; and the large number of irrelevant documents on the web necessitates carefully designed and highly specific search strategy vocabulary [17]. The comprehensive web search steps are described below.

#### Data sources and search strategy

**Fig. 3:**
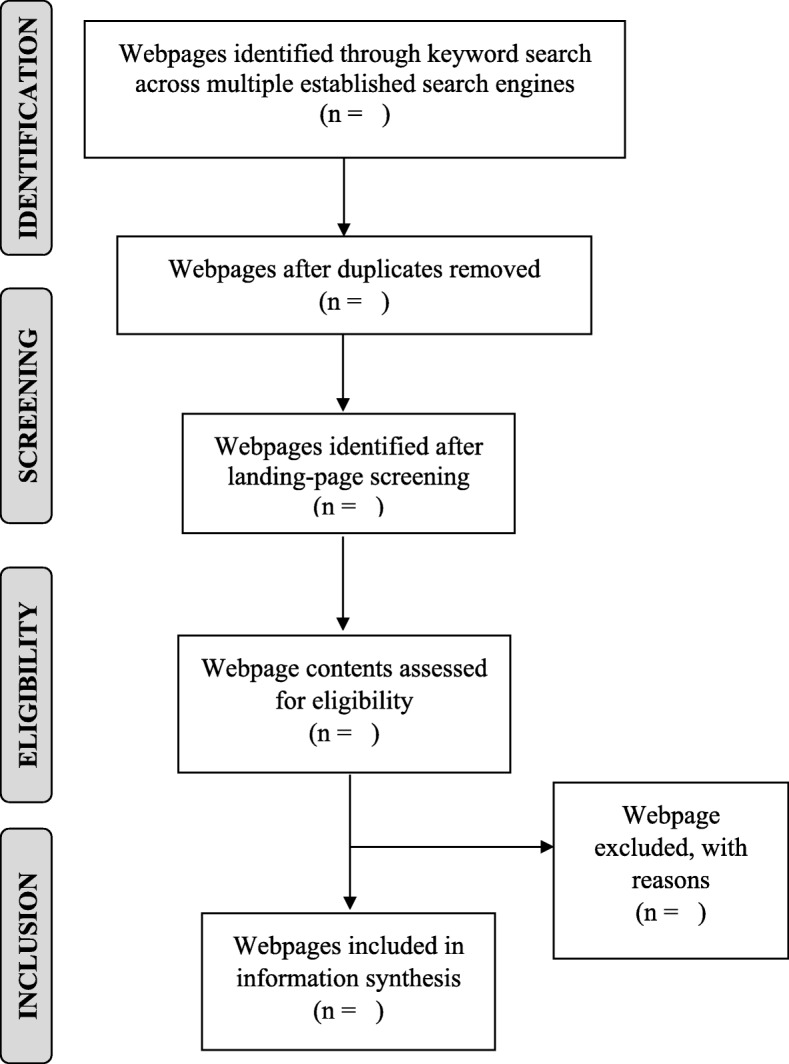
Flow diagram of search and selection process for the comprehensive Internet scan

We will conduct a search (Fig. 3) in a predefined time using the three most popular search engines, Google (Google.com, Mountain View, CA, USA), Yahoo! (Yahoo.com, Sunnyvale, CA, USA), and Bing (Bing.com, Microsoft Corporation, Redmond, WA, USA), as these search engines represent more than 96.4% of all search engines worldwide [18]. In addition, meta search engines that blend web results from Google, Yahoo and Bing, namely, MetaCrawler (Metacrawler.com, University of Washington, Seattle, WA, USA) and Monster Crawler (Monstercrawler.com, London, England, UK) will be consulted as well. The Internet will be used both to identify relevant Web-based information on CBHWLIs and to identify references to non-web-based information. Our web search will include all relevant webpages from government and non-government organizations, news sites, blogs, discussion boards, and social media platforms We will execute Internet searches using the following keyword strings: (1) health/(wellness OR wellbeing) literacy; (2) health/(wellness OR wellbeing) education; and (3) community literacy. Efforts will be made to obtain any relevant documents that elaborate on the development or use of the CBHWLIs. This may involve searching other sections of a multipage website and/or contacting the developers of the tools directly to request further information. Adhering to a methodology utilized by the Canadian Institute for Health Information (CIHI), we will include only the first 10 search engine result pages, consisting of the first 100 results, to conduct a comprehensive search [19].

#### Webpage selection and inclusion criteria

The purpose of the comprehensive webpage search is to capture all relevant documents, webpages, blogs, news sites, etc. that are not covered in the published and grey literature using wide definitions of terms to ensure broad coverage. We will assess the credibility of the webpages based on the criteria recommended made by the National Network of Libraries of Medicine [20]. We will not restrict our search based on country of origin or the scale of initiatives. We will limit the search to the English language only. We also plan to include those sites that are in other languages but have an English version. From the resulting websites, we will exclude duplicate websites, websites targeting healthcare professionals, pharmaceutical websites, sites targeting specific disease-related patient populations, and sites with non-functioning links. To supplement these searches, we will consult with the review team members, content experts, and stakeholders to suggest additional sources they feel would be important to include in our review.

#### Webpage screening

Owing to the dynamic nature of the Internet, the screening and full review of webpages identified in the searches will be conducted concurrently. The primary reviewer will perform the first search string, archive the link-list of the first 100 results (i.e., web address, page title, brief description, and date searched) and classify each as potentially useful or not useful for our objective based on the information available on the landing page. The reviewers will then open all pages considered potentially useful and assess the full webpage content for inclusion. Reasons for exclusion will be recorded. Following the process described by Donnelly and Thompson [21], one researcher will independently assess random selections of 10% of the websites classified as eligible (included) and 10% of those classified as ineligible (excluded).

#### Data abstraction and presentation

Search results will be managed in EndNote software (Clarivate Analytics, Philadelphia, PA, USA). After duplicate entries are removed, we will collect the executive summary or any description provided in all websites identified. We will use a predefined checklist to abstract information from the webpages.

### Key informant/expert interviews

Based on the findings of our scoping review and Internet search, a purposive snowball sampling technique [22] will be used in order to include a wide variety of key informants whose input would be central to the objectives and conduction of a CBHWLI. Our approach in this phase is described below.

#### Identifying key informants through stakeholder analysis approach

Based on conversations with our internal project team, including the community-based citizen researchers, and findings from the scoping review and Internet scan—we will develop a list of key informants who would be important to reach out to for CBHWLI focusing on immigrant communities. These informants will be able to contribute a diverse and complementary set of perspectives. Potential informants will be identified using the following criteria:

- Actively working in community initiatives,
- Actively involved/key management role in health and wellness initiatives of different formats, and/or
- Actively involved health wellness implementation research and knowledge mobilization.

#### Identifying and recruiting participants

We anticipate that our key informants will include community development champions, general educators, health and wellness professionals/academics, health and wellness educators, and leads of organizations working with immigrants. The identified individuals will be informed about the study via email or telephone and will receive a follow-up email if they do not respond within two weeks. In order to carry out in-person key informant in-depth interviews, we will develop a semi-structured interview protocol based on our guiding questions (Table 2). Further, the interviews will allow us to obtain feedback on the effectiveness of these initiatives, culminating in priority areas that could make a difference. The interview protocol will outline the purpose of the study, describe the structure of the interview, outline the consent to participate, and provide some example questions to discuss during the interview. Upon agreeing to participate, an in-person interview will be scheduled.

#### Interviews

The interviews will explore participants’ experience or perception of carrying out of a CBHWLI in practice, the factors that promote or inhibit these programs, the skills and competencies required to carry out these initiatives successfully, and any areas where they feel a CBHWLI is more difficult to put into practice. Trained research team members (interviewer and note-taker) will conduct the interviews. Each interview will involve stakeholders with different skill sets that are relevant to such initiatives. At the beginning of each interview session, the participant will be fully informed about the purpose of the project, the procedure for data collection and how discussions will be conducted. Participants will be clearly informed that they may choose not to participate or quit at any time during the session. These interviews will follow a semi-structured interview approach, whereby researchers and participants engage in a dialog guided by customized questions. Furthermore, researchers will ask follow-up, clarifying and specifying questions about important comments that surface in the conversation. Similarly, participants will be able to add comments they deem relevant to the conversation and to contribute their own perceptions on CBHWLI.

Each discussion will be audiotaped, with permission, using a digital recorder to maximize data collection and assist in transcribing discussions for analysis. Transcription will be done in Microsoft Word and will then be transferred to NVivo (NVivo, QSR International, Melbourne, Australia, or HyperRESEARCH, Researchware, Inc., Randolph, MA, USA). Confidentiality of data will be guaranteed by (1) storing all interview data in a password-protected computer; (2) ensuring the interviews are not linked to any identifiable information to the key informants.

#### Data analysis and presentation

A research team member will compare and merge the field notes taken during each interview to create one comprehensive document. Data analysis will include both the transcript and field notes. To produce a set of themes on the issues and priorities considered important by participants, interview data will be analyzed using an inductive thematic analysis technique outlined by Braun and Clark [23]. In an inductive approach, the themes identified are strongly linked to the data and the process of coding occurs without trying to fit the data into a pre-existing theory or framework. This analysis technique will allow for identifying, analyzing, and reporting patterns (themes) within data, which is done by the following phases. The first phase involves data familiarization, where the transcribed data will be read to note down initial ideas. The next phase involves generating initial codes. Here, two data analysts will code each transcript independently highlighting features of the data that correspond with our research question. Each transcript will be coded in detail, developed through iterative reading. The next phase will involve generating a thematic map, where the two data analysts will consult with each other to organize all codes into comprehensive themes. This is followed by the next phase, which would result in generating clear definitions and names for each theme, through ongoing analysis and refining of the coded data. Finally, the last phase involves the production of a result outlining the themes that emerged from the interviews, accompanied by compelling extract examples from interviews, and concepts from literature. Throughout the interview process, coding, analysis, and synthesis, we will try to be reflective about the various perspectives each of us (academic, community champion, administrator, leader, etc.) brought to the research.

To confirm themes extracted from the data, we will conduct member checking [24] whereby we will share the summary of the transcript with each participant to ensure the explanations and interpretations of the participants’ accounts reflect their views. When presenting information gathered from the interviews, key informant names will not be disclosed in any published materials.

### Community consultation through engagement

After the thematic analysis of the interviews from the previous step is completed, along with the results from our literature and web search, we will initiate consultations with immigrant community members in Calgary, as they represent the population of interest, to help us understand their thoughts about the CBHWLI. They will help us identify factors that matter to them in approving or opposing the idea of impactful and sustainable CBHWLI. We also wish to understand whether they actively want to engage in CBHWLI. We will also try to identify their knowledge needs in case they express a desire for more information while making an informed judgment on the CBHWLI. This will help us understand the external and internal resources, facilitators, and constraints in implementing a CBHWLI. The steps for this section are described below.

#### Community engagement for recruitment

Our objective is to identify grassroots community members’ perspectives (knowledge, experiences, perceived needs, attitudes, and beliefs) of a CBHWLI and to understand how the community can be engaged in discussions regarding the advancement of existing programs or development and implementation of impactful CBHWLI programs. Meaningful engagement of immigrant community members is thus essential for the success of this component. We plan to approach the immigrant community champions or leadership to circulate the invitation for participation to their community members. We will conduct 20 focus group discussions (10 for men and 10 for women, each with 7–10 participants). Individuals who respond to the invitation will be informed about the study via email and telephone. We will develop a semi-structured interview guide focusing our guiding questions and our insights from the previous three steps. The discussion protocol will outline the purpose of the study, describe the structure of the discussion, outline the consent to participate and provide sample questions to be discussed during the conversation. We will also collect individual-level descriptors, including the socio-demographic characteristics, of the focus group participants.

#### Focus group discussions

Focus groups are an appropriate method for this topic because they allow participants to express issues that are important to them [25, 26], which can then be used to generate deep insight into how the participants feel about the proposed CBHWLI as a community empowerment and health and wellness promotion initiative. The discussion will explore participant views on carrying out a CBHWLI in practice, including both the process and documentation, the factors that promote or inhibit a CBHWLI, and any areas where they feel a CBHWLI is more difficult to carry out in practice. We aim to incorporate the difference in gender, age groups, length of stay in Canada, and education level while arranging the focus groups. Also, we plan to organize the focus groups based on the commonality of the language spoken. Trained bilingual moderator and note-takers will conduct the focus group discussions and will ask questions, listen, keep the conversation on track, and ensure everyone in the group has a chance to share their views. Each focus group will last for 60–90 min. At the beginning of the discussion, participants will be advised of the objective of the discussion, and voluntary informed consent will be obtained. Participants will be informed clearly that they may choose not to participate, skip any question, or quit at any time during the sessions.

#### Data transcription, analysis, and presentation

Each session will be audiotaped with permission from the participants. All discussions will be transcribed verbatim, de-identified, and imported into qualitative data analysis software (NVivo, QSR International, Melbourne, Australia, or HyperRESEARCH, Researchware, Inc., Randolph, MA, USA). Data will be analyzed thematically, similar to the process used for individual interviews [27]. Each focus group will be coded individually and themes merged later, as appropriate. Confidentiality of data will be guaranteed by (1) storing all interview data in a password-protected computer; (2) ensuring the interviews are not linked to any identifiable information.

#### Data analysis and presentation

The data analysis and presentation process will follow that undertaken for the key informant/expert interviews previously described. Specifically, the process will involve (i) comparing and merging field notes; (ii) data analysis of both the transcript and field notes; (iii) identifying, analyzing, and reporting themes within the data; (iv) coding each transcript; (v) generating a thematic map; (vi) generating definitions and naming themes; and (vii) outlining the themes and providing examples from the interviews. We will maintain the same reflective process throughout the interviews and coding, analysis, and synthesis. Again, participant names will not be disclosed in any published materials.

## Ethics approval

We intend to publish the results of the environmental scan in academic as well as non-academic outlets to contribute knowledge about CBHWLIs. The systematic scoping review and Internet scan components will not need an ethics committee approval. We will seek institutional ethics approval for the key informant interview and community consultation components as we intend to ensure informed consent to participate, approval to record the conversations, and permission to reconnect with the participants in the steps of result synthesis and summarization.

## Discussion

## Anticipated outcomes

This environmental scan will identify and assess existing CBHWLIs focusing on immigrant communities as the first step. It will also inform us about the barriers and facilitators that have affected the impact of those initiatives. In addition, it will capture the perceptions of key stakeholders and grassroots community members on an impactful CBHWLI. It is an important first step towards developing a guideline for improving or establishing a CBHWLI. We will deliver evidence-based recommendations for future programmatic implementation work on CBHWLI. These recommendations would contribute towards community development, especially in the immigrant communities. This would subsequently improve the quality of community health by assisting immigrant community members to be more involved in managing their own health and wellness needs and become their own advocate for the betterment of life by being active meaningful partners of the health and wellness initiatives in the society.

### Strengths and limitations

A key strength of this environmental scan protocol is the integrated knowledge translation approach and the use of a comprehensive methodological framework to answer the research question. This approach allows us to maximize the potential of knowledge engagement and mobilization at the community level. Also, a team of experienced researchers is undertaking the work. The team includes a librarian (MV) with experience conducting systematic academic, grey, and web searches, who assisted in crafting the search strategy. We plan to apply a comprehensive data extraction template and to use a flexible approach for data acquisition and synthesis. The citizen researchers and community activists were part of the working group from the brainstorming phase of this study. Their involvement gave us the opportunity to co-develop this endeavor.

Notwithstanding the strengths, there are few challenges we need to keep in mind while accomplishing this protocol. First, given the complexity and breadth of definitions for health and wellness literacy and the program implementation structure, careful consideration needs to be given to ensuring the best evidence is identified to answer the research question. A flexible approach to search terms and keywords is necessary to ensure more studies are identified for review. Nevertheless, this study is the first step in establishing a practical base for developing a strategic approach of CBHWLI focusing on immigrant communities ensuring community-centered impact.

### Integrated knowledge mobilization

Our integrated knowledge translation strategy will employ a core philosophy and mechanisms for engaging end-users in the research process and dissemination and implementation of findings, drawing on the Ottawa Model for Research Use [28–30]. We already have actively engaged community member citizen researchers and a multisectorial team from the inception of this idea. This will also help us to disseminate the findings to the policy-makers, appropriate stakeholders as well as at the grassroots community level by creating appropriate info-graphics, pamphlets, and posters with the guidance of our citizen researcher team members. We will also broadcast our findings in lay terms targeting the community members through social media, ethnic language newspapers, or ethnic online news outlets, or placing the knowledge translation materials at social events. We will be doing this in every step of the project as a part of our maintaining continuous engagement with the community. Through doing so, we hope to enhance all levels of participation relevant to the next steps towards improving health and wellness literacy among the immigrant communities. It is hoped that the environmental scan will facilitate future directions and potentially identify improved mechanisms for more targeted health and wellness literacy programs.

## References

[1] Lee HY, Rhee TG, Kim NK (2015). Health literacy as a social determinant of health in Asian American immigrants: findings from a population-based survey in California. J Gen Intern Med.

[2] Rootman I, Gordon-El-Bihbety D (2008). A vision for a health literate Canada.

[3] Hasnain-Wynia R, Wolf MS (2010). Promoting health care equity: is health literacy a missing link?. Health Serv Res.

[4] Suri VR, Majid S, Chang Y-K (2016). Assessing the influence of health literacy on health information behaviors: a multi-domain skills-based approach. Patient Educ Couns.

[5] Sudore RL, Yaffe K, Satterfield S (2006). Limited literacy and mortality in the elderly. J Gen Intern Med.

[6] Lamanna F, Lenormand M, Salas-Olmedo MH (2018). Immigrant community integration in world cities. PLoS ONE.

[7] Mantwill S, Schulz PJ. Does acculturation narrow the health literacy gap between immigrants and non-immigrants—An explorative study. Patient Educ Couns 2017;100(4):760-67]. 10.1016/j.pec.2016.10.021 27856066

[8] Reitmanova S, Gustafson DL. “They can’t understand it”: maternity health and care needs of immigrant Muslim women in St. John’s, Newfoundland. Matern Child Health J 2008;12(1):101-11 %. 10.1007/s10995-007-0213-4 17592762

[9] Berkman ND, Sheridan SL, Donahue KE (2011). Low health literacy and health outcomes: an updated systematic review. Ann Intern Med.

[10] Shahid M, Turin TC. Conducting comprehensive environmental scan in health system & policy research: A process for assessing the subject matter landscape. J Biomed Analytics 2018;1(2):71-80. doi: 10.30577/jba.2018.v1n2.13.

[11] Arksey H, O'Malley L (2005). Scoping studies: towards a methodological framework. International Journal of Social Research Methodology.

[12] Samnani SS, Vaska M, Ahmed S (2017). Review typology: the basic types of reviews for synthesizing evidence for the purpose of knowledge translation. J Coll Physicians Surg Pak.

[13] Levac D, Colquhoun H, O'Brien KK. Scoping studies: advancing the methodology. Implement Sci;5(1):69. 10.1186/1748-5908-5-69 PMC2954944 20854677

[14] Ahmed S, Vaska M, Turin TC (2016). Comprehensive systematic search process of health literature: hunting pearls out of the sea. JNHFB.

[15] King’s College London. Library guides. Searching for systematic reviews: advanced search techniques. https://libguides.kcl.ac.uk/systematicreview/advanced [accessed September 26 2019].

[16] Vaska M, Chowdhury MZI, Naidu J, Baig K, Turin TC (2019). Exploring all that is Grey in the health sciences: What is Grey Literature and how to use it for comprehensive knowledge synthesis. JNHFB.

[17] Eysenbach G, Jadad AR. Evidence-based patient choice and consumer health informatics in the Internet age. J Med Internet Res 2001;3(2). 10.2196/jmir.3.2.e19 PMC1761898 11720961

[18] Alsaiari A, Joury A, Aljuaid M, et al. The content and quality of health information on the internet for patients and families on adult kidney cancer. J Cancer Educ;32(4):878-84. 10.1007/s13187-016-1039-9 27130549

[19] Canadian Institute for Health Information. Urban physical environments and health inequalities. Literature search methodology paper. Ottawa, ON: CIHI, 2011.

[20] National Network of Libraries of Medicine. Evaluating health websites 2015 [Available from: https://nnlm.gov/outreach/consumer/evalsite.html accessed 2020-02-29.

[21] Donnelly KZ, Thompson R. Medical versus surgical methods of early abortion: protocol for a systematic review and environmental scan of patient decision aids. BMJ open;5(7):e007966. 10.1136/bmjopen-2015-007966 PMC4513513 26173718

[22] Marshall MN (1996). Sampling for qualitative research. Fam Pract.

[23] Braun V, Clarke V (2006). Using thematic analysis in psychology. Qualitative research in psychology.

[24] Krefting L (1991). Rigor in qualitative research: The assessment of trustworthiness. Am J Occup Ther.

[25] Morgan D, Scannell AU. Planning focus groups. In: Morgan DL, Krueger RA, eds. The focus group kit. Vol. 2. Thousand Oaks, CA: SAGE Publications, Inc., 1998:1-139. 1998.

[26] Bertrand JT, Brown JE, Ward VM (1992). Techniques for analyzing focus group data. Eval Rev.

[27] Krueger RA, Casey MA. Overview of focus groups. In: Focus groups: a practical guide for applied research. 3rd ed. Thousand Oaks, CA: SAGE Publications, Inc., 2000:3-19.

[28] Estabrooks CA, Thompson DS, Lovely JJE (2006). A guide to knowledge translation theory. J Contin Educ Health Prof.

[29] Shommu N, Choudhury SR, Turin TC (2017). Knowledge translation in health care: bridging the gap between “knowledge generation” and “knowledge implementation”. JNHFB.

[30] Graham ID, Logan J, Harrison MB (2006). Lost in knowledge translation: time for a map?. J Contin Educ Health Prof.

